# The lived experiences and perceptions of middle-aged adults in Dominica who have survived severe storms—a qualitative exploration

**DOI:** 10.3389/fpsyt.2024.1372971

**Published:** 2024-06-04

**Authors:** Josie-Ann LeBlanc, Waneisha Jones, Heather Harewood

**Affiliations:** Faculty of Medical Sciences, The University of the West Indies, Bridgetown, Barbados

**Keywords:** severe storms, Dominica, SIDS, psychological resilience, disaster preparedness, self-help, Eastern Caribbean

## Abstract

**Introduction:**

The Eastern Caribbean island of Dominica has experienced diverse negative effects from the North Atlantic hurricane season, including deadly storms like Hurricane Maria in 2017. Vulnerability is increased by geographic location, small island developing state (SIDS) status, and ecosystem characteristics. A variety of negative health effects including stress and anxiety are caused by powerful storms. The perspectives of middle-aged (the “sandwich generation”) survivors in this post-storm milieu are understudied.

**Methods:**

This phenomenological qualitative study describes the perceptions of middle-aged (35–55 years) Dominicans, purposively recruited with gatekeeper assistance from communities stratified according to four natural hazard vulnerability categories designated by the Climate Resilience Execution Agency for Dominica (CREAD), regarding their lived experiences in the context of severe storms. Data was collected between June and August 2022, using primarily Zoom-based semi-structured, individual interviews (12 of 13), guided by the principles of saturation and maximum variation. Verbatim interview transcripts were thematically analyzed with constant comparison using an ATLAS.ti-supported hybrid deductive-inductive coding frame. Reflexivity and contact summary sheets were used to minimize bias.

**Results:**

Ten women and three men from diverse CREAD vulnerability and sociodemographic backgrounds were recruited. Data condensation yielded three organizing themes: (i) “The diverse health effects of severe storms”, (ii) “Response to and recovery from severe storms”, and (iii) “Preparedness and precaution for severe storms”. These themes encapsulated the health impacts of severe storms on Dominicans and elucidated the role of facilitating and barricading resilience factors.

**Discussion:**

Severe storms produced direct and indirect mental, social, and physical health impacts on middle-aged Dominicans, including anxiety and burnout. Participants used faith-based, tangible community-based support, and emotional mechanisms to cope with and demonstrate resilience. Better risk communication and early warning systems would improve population readiness. Persistent dispirited attitudes toward storm preparedness among some participants suggest the need for targeted methods to enhance community involvement in disaster planning, including traditional approaches like “coup-de-main” (self-help).

## Introduction

Disasters are catastrophic disruptions to a community’s functioning that exceed its ability to manage using only its own resources (1). This paper considers the health-related impacts of hurricanes and other major tropical weather events, collectively described as severe storms, in the context of Eastern Caribbean Small Island Developing States (EC-SIDS), using the Commonwealth of Dominica, a middle human development index (HDI) island, as a case study. SIDS are described by the United Nations as having economic and social vulnerabilities inherent in their small size (2). Typically Caribbean, Dominica has a mixed religious profile incorporating European traditions and Pan-African ideologies (3). Christianity is most prevalent, practiced by more than 90% of the population, mainly as Roman Catholicism and evangelical Protestantism. Non-Christian religious groups include Rastafarianism, Islam, and the Baha’i faith (4).

EC-SIDS are located directly within the North Atlantic hurricane belt, rendering them susceptible to storms every “hurricane season”, which officially runs from June 1 to November 30 every year (5). The relevance of the pervasive vulnerability to severe weather events is clear because over 30% of the countries with the highest annual natural disaster losses are SIDS (5). Furthermore, when estimated as a ratio of GDP, the average effects of natural disasters is 4.5 times higher generally for small states, but six times higher specifically for Caribbean small states (6). Caribbean SIDS experience average annual losses equivalent to 17% of GDP from severe storms (6).

Dominica has experienced over 15 major weather systems since 1950, including over 11 hurricanes (6). Most recently, Tropical Storm Erika (2015) and Hurricane Maria (2017) impacted the island within a relatively short 2-year span, causing remarkable damage to infrastructure, disruption in livelihoods, population displacement, and loss of life (7–9). Dominica’s challenges to recover after repeated “hits” from these natural disasters have been highlighted in regional and international fora (6, 8). Climate change heightens the vulnerability of EC-SIDS due to likelihood of more frequent and destructive storms (5, 6, 10), and the ensuing primary and secondary effects on human health and the environment (6). Primary effects arise from the immediate direct impacts of storms such as wind and rain and include damage to homes and crop losses, whereas secondary effects are ensuing indirect consequences arising from the primary event and may be immediate onset such as displacement from homes or may develop over time and include the social impacts of disrupted daily lives (6, 7). Collectively, these consequences of adverse weather systems can worsen existing vulnerabilities or create new concerns for affected communities (8–10).

Severe weather exposes people to ongoing, resurgent, and novel environmental stressors that may have significant and wide-ranging negative influences on health (11). Hence, planning and preparing for severe storms is a priority of public health and disaster management agencies in the Caribbean (12), embodied in the disaster management cycle, the process by which governmental agencies and civil society prepare for, mitigate, and respond to disasters, and coordinate post-disaster recovery efforts (13). Governmental interventions include risk communication, delivery of healthcare services, and provision of emergency shelters for residents whose homes are considered vulnerable or who become displaced after a storm (7, 12). A 2019 study in the West Indies recorded that over 50% of people who came to hurricane shelters came for health-related reasons such as treatments or consultations (12). Apart from physical health concerns, there is a growing need for mental health services after severe weather events due to manifestations such as stress, anxiety, and depression (14).

The post-storm demands on the health and social care system, though relevant, often appear understated and overshadowed by the post-storm imagery of felled trees and destroyed buildings commonly portrayed in the popular media (6, 15). This is reinforced by the historical focus of international development and financial agencies on quantifying post-disaster economic losses (6, 16), perhaps due to its relevance for justifying aid requests. Similarly, multiple studies on severe storms in Dominica have concentrated on the effects on other sectors and industries, including education (17), fishing (18), and vegetation (19), rather than focusing on the health implications. Recently, estimated storm-related damages and losses totaled roughly USD 1.3 billion, or 224% of Dominica’s 2016 GDP (20). This far exceeds the 17% of GDP average loss for Caribbean SIDS. Agriculture suffered most (32%) of the USD 380.2 million in losses, followed by tourism (19%) and transportation (19%) (20). This paper seeks to address the imbalance by capturing health and wellbeing lived experiences of severe storm survivors. This explication of survivors’ expressed needs provides added insight into the overall societal costs of severe storms.

Within the last decade, more recent efforts have studied the secondary effects of Tropical Storm Erika (2015), including the needs of survivors (no specific age group) who were forced to flee homes (7, 10), and investigated sleep hygiene issues (insomnia and sleep disturbances) and religious coping strategies (prayer and spiritual rituals) of young adult (ages 18 to 25 years) survivors (21). Both studies highlighted several problems among the survivors, but neither specifically examined the survivors’ experiences and perspectives regarding their health and wellbeing following the storm. Studies conducted after Tropical Storm Erika (2015) and Hurricane Maria (2017), respectively, captured the lived experiences of Dominicans through a photo essay and via in-depth accounts of lived experiences (7, 8). Nevertheless, there are calls for further research and prioritization of public health interventions related to climate-related events and health within Caribbean SIDS (10, 14).

A further gap is the relative lack of research targeting the health and wellbeing of middle-aged adults (22). Middle age is the phase of the adult lifespan between youth and old age and may be divided into early (35-44 years) and late (45-64 years) stages. Middle-aged adulthood is complex, associated with physical and psychological vulnerabilities, respectively linked to onset of bodily decline and the weight of familial and societal responsibility. Yet, there are positive features such as experiencing the zenith of productivity and social standing (22–25). Busy lifestyles limiting recruitment of middle-aged participants and interest in the perceived lower health-seeking and higher susceptibility to health problems among teenagers and young adults and in the health deterioration of old age have collectively been advanced as reasons for the lower representation of middle-aged adults in the literature (25–27). This age group is relevant to the current study because middle-aged adults can be considered as the linchpin generation in Dominica. As many as two-thirds of middle-aged individuals are responsible for caring for parents, in-laws, minor children who live at home, or grandchildren. They also make up the bulk of the labor force and more than 40% of the population (28) in Dominica. It is foreseeable that there would be increased societal demands placed upon middle-aged individuals in the context of severe storms.

The aim of this study was to address existing knowledge gaps by seeking contextualized data to describe the perceptions of middle-aged Dominicans and to explore the country-specific nuances (29), regarding their lived experiences in the context of severe storms.

## Materials and methods

This phenomenological study, guided by a post-positivist interpretivist paradigm, captured the subjectivity of reality (30) of participants via one-on-one in-depth semi-structured interviews. The study received ethical approval from the University of the West Indies-Cave Hill/Barbados Ministry of Health Research Ethics Committee/Institutional Review Board (IRB Number: CREC-CH.00042/03/2022), and from the Institutional Review Board (IRB) of the Commonwealth of Dominica and was conducted with the consent of participants.

### Setting

This study took place in the Commonwealth of Dominica, an EC-SIDS, with a densely forested, mountainous terrain, incorporating nine dormant volcanoes and many waterfalls and rivers (31). Dominica’s geographical position of 15.4° N and 61.4° W centers it within the middle of the hurricane belt that lies between 20° and 30° latitude (32, 33). In terms of localized susceptibility to severe storms, the Climate Resilience Execution Agency for Dominica (CREAD) has sub-divided the island into four vulnerability levels: “least vulnerable”, “vulnerable”, “more vulnerable”, and “most vulnerable”, based on the Hazard Vulnerability and Risk Classification for Communities (34). Owing to COVID-19 restrictions, all interviews except one (face-to-face, observing all COVID-19 protocols) were conducted via end-to-end encrypted virtual conference communication platforms, Zoom, and WhatsApp, in acquiescence to the participants’ preferences.

### Target population, sampling, recruitment strategy, and data collection

Purposive sampling, with the assistance of gatekeepers (persons with frontline roles within governmental and civil society agencies involved in disaster management and with associated knowledge of communities impacted by severe storms), was used to recruit middle-aged adults, 35 to 55 years, who experienced at least one severe storm while residing in Dominica. The primary investigator (JL, a female Dominican student with master’s level training in qualitative methods) engaged the gatekeepers, sharing the context of the research and the inclusion and exclusion criteria. Gatekeepers shared the study information including the purpose, researchers’ names, researcher contact details, research procedures, and flyers with potential participants and referred potential participants to JL. To obtain a heterogeneous sample, participants were recruited across a range of sociodemographic factors for maximum variation (35). These factors included gender, age, education level, employment status, profession/vocation, household living arrangements, and the CREAD vulnerability classifications. Recruitment was augmented with the use of a flyer, circulated via social media platforms (Facebook, WhatsApp, Twitter, and Instagram), and with a link to a Google form that allowed individuals to register their interest in the study directly to JL who made the final selection of all referred and self-registered participants recruited into the study, based on the inclusion and exclusion criteria. Participation was voluntary and no incentives were offered.

### Data collection

A piloted, semi-structured interview guide, developed by JL and HH (a researcher with doctoral training in qualitative methods, a medically trained public health practitioner with experience working in disaster management activities in a SIDS setting, and JL’s research supervisor) and reviewed by WJ (a medically trained researcher with master’s level training in qualitative methods and JL’s co-supervisor), was used by JL to explore participants’ perspectives regarding their (i) personal experiences of severe storms, (ii) health state before and after the storm, (iii) the effects of severe storms on their lives, (iv) process of recovery after the storm event, and (v) future storm preparedness plans. JL conducted in-depth interviews, averaging 30 min in length, between 20 June and 23 July 2022. Emergence of saturation was noted at the 12th participant at which point JL recruited one more male participant to test saturation and to support achievement of maximum variation. Saturation of themes was confirmed at the 13th participant. JL took field notes, completed contact summary sheets, and debriefed with HH and WJ after interviews to identify, discuss, and clarify emerging concepts and perceived gaps in data coverage. The researchers shared data between password-protected personal use devices using 7-Zip^®^ encryption software.

### Analysis

JL listened to the audio recordings of the interviews immediately upon completion. The recordings were transcribed verbatim. Standard English substitutions for dialect (local creole language) terms used by participants were indicated in brackets. A nine-item hybrid coding dictionary (**Table 1**) was developed first with the derivation of deductive codes based on the concepts used in the interview guide, and later expanded with additional inductive codes developed by JL and HH. All code definitions were discussed, refined, reviewed, and finalized by JL and HH. The codes were subsequently applied independently by each member of the research team to three transcripts, with discussion and resolution of differences. Thereafter, JL completed the coding. Thematic analysis with constant comparison was done, using ATLAS.ti software to facilitate the data reduction process. The research team reviewed and discussed emerging issues and patterns, which were subsequently reduced into basic, organizing, and global themes.

**Table 1:** Coding dictionary with code abbreviations and descriptions.

	Name of code	Abbreviation	Explanation of code—all references by participants regarding…
1	Social Health Impact	SHI	The positive and negative impacts the storm had on the social health of participants/their communities after and/or during the storm(s)
2	Physical Health Impact	PHI	The positive and negative impacts the storm had on the physical health of participants/their communities after and/or during the storm(s)
3	Mental Health Impact	MHI	The positive and negative impacts the storm had on the mental health of participants/their communities after and/or during the storm(s)
4	Coping Mechanism	CM	The instance the participants used different tactics or mechanisms to cope with the storm(s) passage and aftermath
5	Response to Storms—Community	RAS_C	The response the participants had to the storm and the roles they played within their community during and after the storm(s)
6	Response to Storms—Family	RAS_F	The response the participants had to the storm and the roles they played within their family during and after the storm(s)
7	Preparation for Storms	PFS	How participants are now preparing for the hurricane season after experiencing severe storms. This could refer to both personal and interpersonal factors and steps taken
8	Sensory Experiences	SE	The general experience the participants had during and after the storm in regard to what was seen, heard, and thought
9	Subsequent Recovery	CWL	How life changed and is still changing subsequently for the participants and their family/communities after and due to the storm. This could refer to both personal and interpersonal factors and steps taken

### Reflexivity statement

The primary researcher, a Dominican who has experienced a severe storm, and who volunteered with a community-based disaster management civil society organization, used several reflexivity techniques to assure methodological rigor and thus limit the influences of her own practices, bias, and judgement on the research being conducted (36). Three main reflexivity approaches were used, namely, writing of a reflexive account, debriefing, and critical self-reflection (37). Immediately after each interview, JL made field notes, documenting thoughts on the conduct of the interviews and extent of rapport, and considering the potential impact of personal and other biases on data collection. Contact summary sheets were used to facilitate early interpretation of the data while noting emerging concepts based on participants’ descriptions across successive interviews. Debriefing was done via discussion of the sheets with HH and WJ who also reviewed and discussed the field notes, contact summary sheets, and the coding and analysis process, offering alternative views where appropriate as they examined and discussed emerging themes with a view to ensuring that the participant voice was reflected. Recruitment of an additional male participant to achieve maximum variation, in view of the female preponderance, yielded no new themes; the implications of the relative lack of the male voice were considered in the discussion. Finally, as part of the critical reflection process (38) JL, a storm survivor, was cognizant of the potentially delicate and emotive nature of the topic. There was a concerted effort to stay in the neutral researcher role, including limiting verbal and non-verbal acknowledgments as participants recalled their experiences.

## Results

Thirteen participants, 10 women and 3 men, ranging in age from 35 to 55 years and with at least one person representing each of the CREAD vulnerability groups and each parochial district, were recruited into the study (**Table 2**).

**Table 2:** Sociodemographic profile of the study population.

Participant	Sex	Age range	District	Vulnerability 1—most, 4—least	Self-declared occupation
1	Female	35–39	Southwest	Vulnerable (3)	Education
2	Female	50–55	Southeast	More vulnerable (2)	Public sector
3	Male	40–49	North	More vulnerable (2)	Education
4	Male	35–39	West	Vulnerable (3)	IT services
5	Female	40–49	Southwest	More vulnerable (2)	Public sector
6	Female	35–39	Southwest	Vulnerable (3)	Healthcare
7	Female	40–49	Southwest	Most vulnerable (1)	Unemployed
8	Female	40–49	West	Vulnerable (3)	Program manager
9	Female	35–39	North	More vulnerable (2)	Banker
10	Female	40–49	West	Vulnerable (3)	Education
11	Female	40–49	East	Least vulnerable (4)	Education
12	Male	40–49	South	Least vulnerable (4)	Religious leader
13	Female	40–49	Northeast	More vulnerable (2)	Education

The initial data reduction process yielded 10 basic themes, which were further condensed to generate the three organizing themes (OTs): (i) “The diverse health effects of severe storms”, (ii) “Response to and recovery from severe storms”, and (iii) “Preparedness and precaution for severe storms”, and one global theme (GT): “The physical, mental, and social health impact of severe storms on Dominicans is modulated by facilitating and barricading disaster preparedness and resilience factors” (**Table 3**).

**Table 3:** Data reduction: derivation of the global theme from organizing and basic themes.

Basic theme	Organizing theme	Global theme
- The mental health of participants was affected by sensory experiences they encountered. The sounds, sights, and experiences during and after the storm brought out different mental health impacts in the participants such as fear and anxiety. - The social health of some participants affected their mental health and *vice versa*. For instance, individuals who had good social support tended to have a better mental health outlook and *vice versa*. - The physical health of the participants affected their mental health as well. For some individuals who were distressed mentally, it eventually took a toll on their physical bodies.	The multi-faceted health impacts of severe storms	The impacts that severe storms have had on the lives of middle-aged Dominican are complex interactions between the direct and indirect barriers and facilitators of health and disaster management that affect their lives in the long term.
- The roles the participants played in their communities and families in the context of the hurricanes helped with subsequent recovery. - Participants recovered from health issues and economic issues through the use of different coping mechanisms such as adapting to the new situations. - Different sensory experiences faced by the participants forced them to play roles such as a supporter and a protector to family and community members.	Response to and recovery from severe storms	
- General precaution and safety measure the participants used to prepare for storms both before and after experiencing severe storms.	Preparedness and precaution for severe storms	

The OTs will be used as a framework to present the findings.

### OT 1: “The multi-faceted health impacts of severe storms”

This theme summarizes participants’ reports of how their health was influenced by severe storms and how the storm experience affected their behavior during and after the storm. Multiple effects encompassing mental, social, and physical aspects of health were described.

#### Mental health impacts

Sights, sounds, and other sensory stimuli during and after the storm elicited anxiety and fear within the participants. One participant explained that her experience with the rain and wind evoked fears about the stability of her affected house and concerns for family members’ wellbeing during the storm.

“…*And you know, trying to keep them calm. And you know, people are messaging, you know, what’s going on, you know, you’re trying to be…. half honest, because you don’t want to scare them because at this point, we started getting scared because it felt like the house was shaking, there was just endless rain, we were getting where you were flooded. In the room, we had, you know, there was four or five children, two adults, two women, no men in the house. So, we didn’t have the manpower to do anything to secure anything, or we didn’t even think it was gonna be this bad*.” (Participant 7, female).

Men also experienced fear; this participant described hearing noises and feeling a vibratory sensation, which raised concerns about the stability of the house and ultimately for his survival.

“*Couldn’t see what he could hear like. Like you, swear say like [you would swear that] houses, ripping apart … things flying, hitting de houses, you know? Houses like where I was…, were, was the house was kind of shaking on pillars and like I would say that was the end of my life.*” (Participant 4, male).

Another participant spoke about being so overwhelmingly scared that she uncharacteristically “broke down” during the storm.

“*But the sound of the wind, it was very, very frightening, and troubling. That was my problem. And I think somewhere along the line, I broke down. I really, really broke down. And I started crying. And my husband is there saying to me, “No, you cannot break down. Because if you break down what is going to happen to me?”. And he’s like saying “but you are the stronger one when it comes to these things”. But I just I did … wind was so terrifying*.” (Participant 10, female).

A common thread that emerged within this theme was that the emotional responses seemed to be linked to participants being surprised by and hence apparently unprepared for the severity of the effects experienced.

#### Social health impacts

Participants shared varied experiences and descriptions relating to the impact the storm had on their social health. Several participants described negative experiences associated with unwelcome changes in living circumstances.

*“[Life after the storm] It changed, it changed completely. Because normally I am … I’m an inside person naturally. So, I didn’t miss any of the outside experiences. But then I have to be forced to. Because the I think like the first two weeks after the storm, the people that were on the immediate side of the apartment, all of us lived together. And then they have, some of their relatives came. And then some other people from the community came. So, it ended up having like 20 of us in this what was a one-bedroom apartment. So, I, I liked my personal space. So that was an adjustment for me. So, even more reasons were like, Okay, I will take it [rather live] in the concretes and the plywood for the door. Like I will take it there instead [over sharing with multiple people]. Right? So, it was in terms of having to be forced to socialize even when I didn’t want to it was a bit straining on for me personally.*” (Participant 6, female).

Conversely, some reported that their social health changed for the better as the storm brought both family and community together. One female participant describes the connectedness she experienced in the recovery period.

“*I kind of enjoyed the sense of community because we would…. what- it brought my family closer in a sense because we would get up really early like crack of dawn like five o’clock in the morning everybody has a cup of coffee or a cup of tea. Then talk about what we going to do for the day. And from the crack of dawn, we would start to work.*” (Participant 11, female).

#### Physical health impacts

Within this study, there were no reported direct personal physical impacts arising from participants’ storm experiences such as injuries. However, activities of daily living were altered for participants due to infrastructural damage resulting in a welcomed increase in physical demands for some. Accordingly, one participant had an overall positive outlook about the new, more physically demanding way of life, presumably due to perceived fitness benefits.

Researcher: “And did that ever take a toll on your physical health?”

Participant: “*Surprisingly, no, I had a lot more energy … oh I forgot what I hated the most was having to wash by hands, that I hated the most. (chuckles) Because the umm- it did not- I was fine I did not it was I even got more muscular. Like well not muscular, lean. Because it was all this manual work and they- It did not really affect me that bad.*” (Participant 11, female).

Alternatively, for other storm survivors, the extent of engagement in manual activity resulted in physical exhaustion and the realization of neglected mental health self-care.

“*Umm, After the storm, I was. (Deep breath) I was healthy. I…. ummm … how should I put it? Because I was in action mode because I had to go back to work. You know, my, my workplace got damaged. So, you had to be moving things. And as I never really got a chance to make mentally, like a mental check. I was just in action mode. And my body was in survival mode. So, we were just go in cleaning and just moving. And it was about a month after that I fell … sick. I just, I fell sick, like I could not move. My body just broke down. I started to feel muscles, the muscles that I never knew what it was. And there was a point in time my mind was like……. I just could not focus properly……And I was it was just I was delusional. I was like, I don’t know, sometimes I will not know where I’m at. Where am I? What is really going on? And then I went to the doctor because I could not walk (chuckles). And he told me that my body overworked itself.*” (Participant 9, female).

In examining the interplay of these health effects, it emerged that mental health was directly influenced by sensory experiences. Conversely, there appeared to be a more nuanced and complex inter-relationship between mental health and aspects of social and physical health, respectively, which appear to be modulated by perceptions about the experiences arising from altered life circumstances after a severe storm.

### OT 2: “Response to and recovery from severe storms”

Participants’ recovery and reaction strategies were affected by individual, interpersonal, and community resources, depending on participants’ access to these particular resources. In describing their coping strategies, participants made explicit and implicit references to emotional and mental wellbeing.

Some participants expressed that they released their emotions privately, using crying to cope with their recent experiences of severe storms. This “lonely” experience, characterized by a persistence of negative emotions, was not limited to persons who were physically alone or isolated.

“*I started getting depressed me alone home. My husband is at work. My neighbors doesn’t really have a home amm … for him to stay you know, but he was in the health center [hurricane shelter]. And…. like things, I would just sit, and I would just cry without even like, saying, well, okay, the tears just used to come. And then I used to get scared because of the silence around me and so on, even though people were in the village, but the silence was deafening, it was frightening.*” (Participant 10, Female).

Contrastingly, other participants used the collective experiences of their social circle to push them towards what appeared to be more effective coping strategies.

Researcher: “And what were the ways that you did try to cope?”

“*Umm … Interacting with friends as one…. Board games…. you know because you don’t have light [electricity] you do have access to certain things. Yes, so your circle my circle, that helps because you know we get to interact, we get to share experiences.*” (Participant 13, Female).

“*But fr- there were friends, we wo- came together. Some of them would come every every two nights we would cook. We would laugh (laughs). We would, we’d tell stories. And that, that was therapy. For, for many months I was therapy. And to this day we remained, we remained friends. So, it helped a lot.*” (Participant 12, male).

Yet, for other participants, the shared experience was at a spiritual level, in that many also used their faith and trust in God to get through the storm and the period afterwards. Participants recounted how they used prayer throughout the storm to encourage others and themselves.

“*And everybody coiled up on the bed and I have the mattress there supporting them. And then, water in my feet and the lady’s asking me “What is happening? What is hap-” I tell her “Just be quiet and just pray.*” (Participant 2, female).

“*And managing, managing grief is (scoffs) well prayer and communication talking to friends. Family members…. prayer, prayer and communication. That’s how you, we manage grief. So that’s what I did.*” (Participant 12, male).

Regarding the post-impact period, participants illustrated how they came to assume roles of responsibility within their family circle, encompassing meeting financial and basic needs for daily living and providing emotional/mental and social support to their social circles. For some participants, synergistic family dynamics made the process manageable.

“*Well, I didn’t lose any loved ones, but a fair- of my relatives lost their homes. And as I said, my aunt lost her- house got damage and other uncle house that damaged they all moved into it with my mom [and I] while they were rebuilding their homes. And ummm, my family is one if one is hurt, all of us is hurt. So (hard breath) we we just pull our resources together to help each other. And by that we all survived through through the help of and support of family.*” (Participant 9, female).

Nevertheless, this emergent responsibility for others appeared to be burdensome for some participants and was consequently perceived as a source of stress.

“*Because in terms of the number of us who were in the house. It was like four of us. But I had like an elderly … My father, who was who strong enough but was not very interested in going out. And my stepmother who could go out, but you know, had few hesitations. So, like I felt all of a sudden, I have to be responsible for these people. How am I going to feed them. What am I going to do? Where err, are we going to get gas [petroleum], how we going to move? That you know that kind of thing.*” (Participant 1, female).

Added to the social responsibility was the physical effort involved in traveling long distances to procure supplies. Although one participant mentioned looting, the statement seemed more of a matter-of-fact description of post-storm activities, rather than an expressed fear or perceived source of psychological stress.

“*It had me thinking, thinking. Like I was worried too, you know, because….my family we didn’t (have) food. So we had to, had to make a way like….Leave from where I was to right down to go … Down all the way to town, on a bicycle because vehicle wasn’t able to pass [roads were blocked]. You know, a lot of walking, hiking, you know. Looting.*” (Participant 4, male).

Many of the described coping strategies and emergent family and community roles influenced some of the participants to change the way they currently prepare for severe storms.

### OT 3: “Preparedness and precaution for severe storms”

This theme summarizes participant’s descriptions of their approaches/attitudes to severe storm preparedness, in the context of being severe storm survivors. Approaches ranging from heightened preparedness to resignation and even avoidant behaviors were being planned. Whereas all participants reported stocking up on non-perishable foods, a mixed picture emerged regarding preparedness activities related to housing and other infrastructural matters. Some participants took a more cautious approach, using previously identified gaps to develop new approaches to storm preparedness, which afforded the opportunity to “build back better”.

“*Well, from the time its hurricane season is approaching you know, the groceries and dry goods and the canned goods and the water and extra medication … And umm now, I am, I’m personally building my own home as at a higher, in an area that is considered to be less prone to disasters, I mean, there doesn’t there’s not much of that in Dominica, but considering we don’t I don’t have to worry about the river and all of that.*” (Participant 6, female).

“*Well, the plan really is that … to always have our food ready our dry foods and whatever ready. But thankfully while I was in the States, I was able to take advantage of some free shipping initiative. My friend umm, suggested it to me and … I bought shutters … for the house. So, they’re already installed. They’re already installed so, it’s just a matter of … getting them down and locking them off if a hurricane is coming.*” (Participant 3, male).

In addition to making provision to protect themselves and their property, some participants also used faith in God as a buttress against worry.

“*Well, the one thing we need to stock up on, we stock up on food and water. That’s for sure. Umm, ensure that the is insurance is this really paid off is after Maria, you you heard to horror stories of insurance, and not being able to cover the replacement value of the house and other things were to make sure just to make sure that was adjusted, and everything is okay. And then stock up on food and water. Because I think at that point, we, that was where I guess we were, because we had just moved, I suppose. Umm … You know, the house wasn’t fully stocked as … should have been. So that is, that is one thing once hurricane season started then you start stocking up.*

Researcher: “Are you concerned about the reoccurring hurricane season?”

(Deep sigh) *This is not really. I mean, when I say not really is not that-, I mean, you’re always concerned that you know, you can get a storm. But honestly, I just think God, is not that wicked. (chuckles) ……Seriously. God is not that wicked to have us go through that in such a short space of time.*” (Participant 8, female).

In contrast, other participants presented a more dispirited outlook and seemed locked into a prolonged contemplative phase and hence could not advance to activating a preparedness plan.

“*To be honest like I haven’t I, I, I haven’t put any … I haven’t started doing anything, but I have been thinking I’ve been thinking of OK … What can I do? What should I do? Where should I start? I need to start getting things done. I need to start maybe preparing my documents and preparing a bag and that kind of thing so.*” (Participant 1, female).

At the most extreme of the preparedness continuum was one participant who opted for flight rather than facing the prospect of another severe storm.

“*But it was a terrifying experience, you know, you face when you face death, you know, you think of everything and the way these things. And you now … have learned not to fear death so much since I (phone notification) accepted it. So, you know, I am to die tomorrow, the fear wouldn’t, I wouldn’t be as fearful, because, you know, somewhat faced something when I faced it already. And you know, it’s inevitable. But I don’t want to experience (chuckles) a hurricane like this. Anytime I can travel out of the Caribbean the hurricane season (laughs) I’m gonna go. Last year, I was not here for the whole hurricane season.*” (Participant 7, female).

### GT: “The physical, mental, and social health impact of severe storms on Dominicans is modulated by facilitating and barricading disaster preparedness and resilience factors”

**Figure 1:**
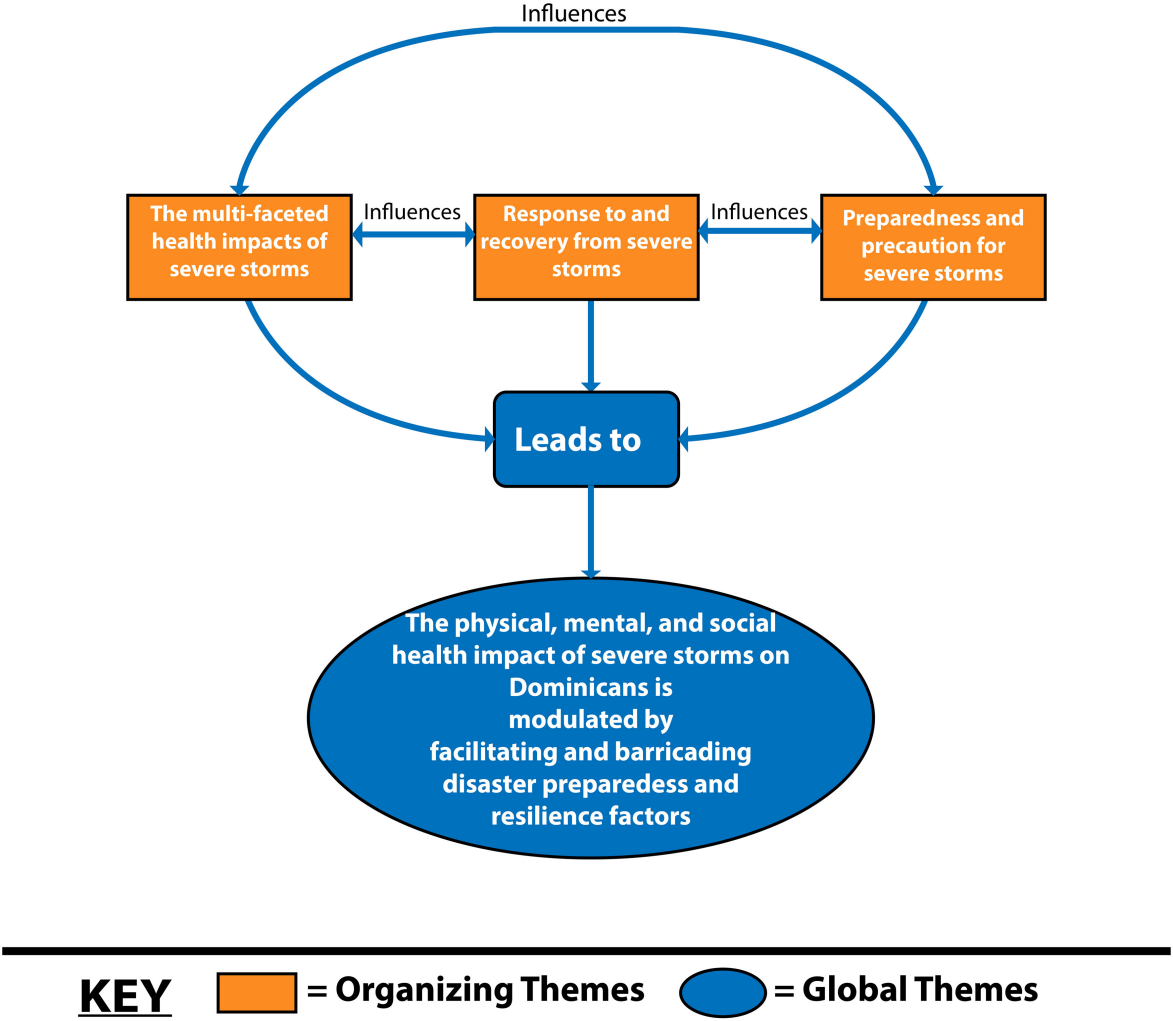
Relationship between organizing (OT) and global (GT) themes.

This combined theme encapsulates the complex interplay of positive and negative aspects of preparedness and resilience factors as they shaped the experiences of Dominicans who experienced severe storms and who remain at annual risk from the Atlantic Hurricane season (**Figure 1**).

## Discussion

The shared perspectives of our participants highlight that severe storms elicit multiple and often interconnected effects on the lives and livelihoods of middle-aged Dominican survivors. This multi-faceted impact was encapsulated in three emerging themes and one global theme encompassing aspects of health, storm response and recovery, and preparedness approaches.

### “The multi-faceted health impacts of severe storms”

The information gathered for this study indicates that middle-aged Dominicans are susceptible to varying direct health effects from severe storms. Hurricanes are known to peak and exacerbate health issues especially 6 months after impact (15). This study was conducted outside this 6-month window, across a wide range of post-storm survivorship (5 to over 40 years) periods but the experiences relayed by participants provided evidence of early onset of emotional distress during the passage and in the aftermath of storms. Despite the length of time between the experience of the storm and the participant interviews, the results illustrate vivid accounts of key concerns related to altered living circumstances, including displacement from homes. Such “trauma” associated with hurricanes can cause previously healthy survivors to develop new mental disorders and aggravate pre-existing mental illness (39). Though no formal diagnoses were made or reported in this study, participants reported feeling anxious, depressed, and fearful in the context of severe storm experiences; this is a common finding among survivors of severe storms (40). Our findings corroborate a recent study that highlighted the emergence of mental health manifestations including fear and anxiety as a dominant feature among Dominicans, after hurricane Maria (41).

The study also highlights that mental health is affected both directly and indirectly by storms, with effects arising acutely in the immediate storm impact (primary) phase and subsequently during the secondary effect phase due to factors such as altered living circumstances. This was one illustration of the interplay between mental, social, and physical health. Displacement from home to new settings has been linked to adjustment challenges. This was documented with internal (rural to urban) displacement in Dominica and with external resettlement to neighboring EC-SIDS (41). External displacement may have amplified negative mental health implications. This was observed among Puerto Ricans relocated to the USA post-hurricane Maria, where elevated rates of post-traumatic stress disorder (PTSD) were observed compared to levels of trauma among survivors who remained in place. This occurrence of increased vulnerability was highlighted as part of the rationale for a comprehensive mental health policy focus within the region (14, 42). Several studies (USA and England) demonstrate higher levels of mental health effects including PTSD linked to internal displacement (43–45). While there is evidence of diminution of these effects over time (45), this has been linked to enhanced mental health service provision. Apart from imposing a more dire initial risk profile for mental ill health in the post-emergency phase, external displacement brings the potential challenge of reduced access to goods and services based on citizenship (41). Displaced Dominicans in Guadeloupe described financial vulnerability linked to unemployment, and limited opportunities for engaging in formal work as a source of stress. There is scope for more robust mixed methods studies to fully quantify and to explicate the nuances of the various displacement scenarios. The health system disaster response should therefore incorporate strategies to address safe sheltering in place, preferably in a familiar community setting. However, use of schools is not recommended (41) due to delayed return to normalcy in the education of children. This, in turn, would impose the added responsibility of remote education by parents and guardians, many of whom belong to the middle-aged range. The physical exertion required in the aftermath to rebuild, coupled with worry about survival and future disasters, negatively impacted some individuals’ physical health. The experience of burnout due to increased physical effort within the context of altered living circumstances, such as lack of the usual modern amenities, is much more likely during mass disasters like hurricanes (40). Notably for others, the additional physical exertion was considered beneficial for their mood and physical appearance.

The demand for mental health services in the wake of large-scale disasters, while unpredictable, is often increased and likely to be challenging in SIDS given resource limitations (16). These resource limitations are an added area of vulnerability from the perspective of governmental provision after disaster. This is because the narrow range of non-human resources and personnel deficits due to migration of skilled workers are well-described health system challenges in SIDS (46). The resource limitations, in turn, limit infrastructural repairs to damaged health facilities and constrain service provision, thus diminishing the availability and quality of health care services after disaster. Furthermore, displaced survivors of Tropical Storm Erika and Hurricane Maria highlighted the inconsistency of mental health services and burnout among healthcare providers (41). Thus, in addition to prioritizing population mental health service provision, psychological support for health providers in disaster contexts is another documented need (47) that should be routinely incorporated into the local disaster planning cycle as part of a comprehensive proactive approach to place care providers to assure the mental health and wellbeing of healthcare recipients and providers. This remains an area of opportunity for Dominica given that the mental health reform process commenced in 2007 (48) did not specify a mental health policy or plan in the context of emergencies. Nevertheless, there is possible scope for increased public–private partnerships based on the current work of non-governmental relief groups such as International Medical Corps, which has been instrumental in providing mental health psychosocial and support services (MHPSS) after storms including Hurricane Maria (49, 50).

### “Response to and recovery from severe storms”

Our participants revealed three broad adjustment pathways to response and recovery, namely, self-management approaches such as private reflection, reliance on spiritual/religious beliefs, and leveraging the collective strength of the community. Based on the descriptions, some of these responses seemed to be reactive coping since they appeared to emerge *de novo* due to the severe storm stressor. Reactive coping is considered adaptive in unpredictable circumstances (51) and may thus be relevant to the climate-induced volatility of the hurricane season in the EC-SIDS setting. Thus, further examination of the coping styles of storm survivors should be undertaken to elucidate the background susceptibility to adverse outcomes within the population and thus inform health system responses to address these vulnerabilities. Based on the contrasting experiences of those who “suffered in silence” versus those who were supported by others, it appears that for overwhelming situations like a severe storm, Dominicans seemed to have better mental health resilience when there was a sense of shared experience and social support.

Mental health resilience, which may be defined as the capacity of an individual to effectively adjust to life’s responsibilities in the face of socioeconomic disadvantage or extremely unfavorable circumstances, is a crucial component in safeguarding and advancing excellent mental health (52). Development of resilience is considered to represent a balance of barricading and facilitating factors (51, 52). It may be argued that social coping (51) as evidenced by the emergent social networks promoted resilience among some Dominicans. A further protective factor may have been the ability to exercise the option to remain in a home-based environment rather than inhabit a formal shelter or face external displacement, the latter of which has been shown in some cases to result in worsened mental health outcomes (41, 53). The described social interactions, including tangible help such as providing room and board for neighbors and meeting the emotional support needs of community members, life partners, and children, coupled with simply having someone to laugh with (emotion-focused coping) (51), helped the participants to deal with the dynamic circumstances being experienced. This has positive implications for informing individual and community mental health interventions because it has been demonstrated that social support, thankfulness, and resilience are linked to greater life satisfaction and fewer depressive symptoms following hurricanes (54).

Emotion-focused coping was further manifested in some participants’ attestations that their faith and confidence in God got them through difficult moments during and after the severe storms. These accounts have parallels with those of survivors of Hurricanes Katrina and Rita in the United States who used spiritual support and humor to cope and become more resilient after the experiences (55, 56). However, it is important to differentiate the favorable religious coping stance inferred from our participants’ statements about a benevolent God to whom they could pray and who was “*not that wicked to have us go through that in such a short space of time*” (Participant 8, female). Arguably this reliant faith in God is bolstered by the manifestations of supporting works undertaken by the various local and regional faith-based organizations (FBOs) to effect post-storm relief on the island. Activities have ranged from provision of meals and advocacy for resources, to rebuilding of homes and places of worship (57, 58). Such “positive religious coping” is linked to good mental health outcomes, in contrast to “negative religious coping” characterized by ominous views of God and associated with adverse mental health outcomes such as PTSD and depression (56). Thus, while many participants used faith, confidence, and emotional support mechanisms to cope and become more resilient, there is scope to further explore what is needed to develop positive skills among persons whose accounts still echoed notes of despair.

In this study, it emerged that participants’ recovery and response processes influenced the way the participants prepared for future severe storms.

### “Preparedness and precaution for severe storms”

Preparedness was enacted in multiple ways for the participants primarily via aspects of objective preparedness (59) such as “applying lessons learnt” from past experiences of being unprepared. This was seen in the form of family disaster plans, preparing hurricane kits, and infrastructural upgrades. This positive change for most should ultimately benefit their health as lower levels of hurricane preparedness are linked to negative impacts on overall health and to a marginally significant impact on non-communicable diseases (NCDs) (60). The role of the Dominican diaspora in supporting community resilience and wellbeing is important because the acquisition of supplies and materials for enhanced preparedness was facilitated by United States-based members of the diaspora who underwrote the costs of the “free shipping initiative” as articulated by some participants. These descriptions are consistent with International Monetary Fund reports indicating significant increases in remittances to the Caribbean in the wake of severe storms (61). Use of emotional coping strategies (62) like a focus on faith to augment preparation for future severe storms appeared to provide additional benefit for some participants. Nevertheless, a lingering concern exists for those individuals who seemed challenged to move toward definitive preparedness action. A lack of association between prior hurricane exposure and improved future preparedness (63) and gaps between subjective and objective preparedness (59) have been identified as important barriers. Being able to execute positive coping mechanisms can help with disaster preparedness and resiliency (64). Further work is therefore needed to explicate the barriers and to subsequently inform tailored interventions to build self-efficacy to implement effective coping and response strategies (60). This is particularly relevant for Dominicans, given the context of an annual hurricane season and impaired cycles of recovery due to repeated severe storm impacts.

Further work is needed to explore the pros and cons of avoidant behaviors in relation to severe storms especially in the context of increasing unpredictability of weather patterns. In some settings, relocating when hurricanes approach or after impact is a “normal” component of hurricane preparedness planning as a protective measure to avert repeated negative experiences (65). In the United States, this can take the form of a permanent move dependent on factors such as income, risk of subsequent disasters, and depth of desire to stay in the current home (64). In the context of this study, only temporary relocation was contemplated. This may reflect the mixed experiences of Dominicans who relocated externally in the aftermath of Hurricane Maria (41). Although this form of preparedness circumvents future lived storm experiences, damage to property left on the island could still occur and may still trigger distress. Therefore, it must be ascertained if the preference to leave represents a problem-focused coping mechanism evidenced by active coping and planning or whether this is a maladaptive avoidant response (51).

## Conclusion

The study’s findings point out many areas where the health system’s policymakers should take action at the various levels of the Social Ecological Model (SEM). The establishment of integrated mental health and social support systems have to be prioritized in order to aid individuals who will potentially encounter severe storms and the ensuing upheavals in their lifetime. This has been articulated on various occasions (10, 14, 41). However, an area for new or renewed scope may be the leveraging of the innate social capital of Dominican society. This draws on the shared experiences of our participants whereby the shared sense of facing the recovery period as a community proved to be a strong coping mechanism. This community spirit and working together, both locally and across the diaspora, is in line with the colloquially termed “coup-de-main” or self-help approaches that have been traditionally used by Dominicans to address various social issues (66) and is supported by the resiliency literature (51, 53). This is relevant as community-based culturally relevant resilience would provide a supportive mechanism for middle-aged people on whom fall the onus for breadwinning, care provision, and contribution to the workforce.

Based on participants’ underestimation of the speed and strength of the severe storms that can affect the island, better risk communication and early warning systems based on all-case scenarios may help improve population readiness. Despite negative experiences, unchanging attitudes toward storm preparedness and safeguards suggest the necessity for specialized methods to enhance community involvement for some sub-sections of the middle-aged population in disaster planning. Future studies might be able to examine the effects of participants’ health on an individual basis or even in terms of the phases of the disaster management cycle.

### Strengths and limitations

The strengths of this study include the involvement of participants from all CREAD vulnerability categories, allowing us to document wide-ranging and variable experiences. In addition, both participant accounts of single and multiple severe storm experiences were incorporated into this study. A further strength is the achievement of data saturation, which was confirmed by the 13th participant. This aligns with the literature, which supports the possibility of achieving saturation between the 9th and 17th participant, particularly when coupled with recruitment to achieve a maximally variable sample, thus enhancing data richness and diversity (67, 68). However, we acknowledge that in the context of a small sample, triangulation with data from documentary analysis and in-depth interviews with other stakeholders such as health providers would have strengthened the study. The study is also limited by the female preponderance; there is scope for future work to further explore the male perspective. While the pragmatic use of the online modality reflected the prevailing COVID-19 restrictions and allowed for mutually convenient, low-risk access to participants (69–71), the potential trade-offs of reduced rapport and lesser ability to assess non-verbal cues are also acknowledged as potential limitations of the study. Finally, the sensitive nature of this research may also have caused people to repress memories and some potential participants felt that the events of the severe storms were too traumatic to discuss or relive and declined involvement in the study.

## Data availability statement

The raw data supporting the conclusions of this article will be made available by the authors, without undue reservation.

## Ethics statement

This study involving humans was approved by the University of the West Indies-Cave Hill/Barbados Ministry of Health Research Ethics Committee/Institutional Review Board (IRB Number: CREC-CH.00042/03/2022) and the Institutional Review Board (IRB) of the Commonwealth of Dominica. The study was conducted in accordance with the local legislation and institutional requirements. The ethics committee/institutional review board waived the requirement of written informed consent for participation from the participants or the participants’ legal guardians/next of kin because the interviews were conducted virtually. Each participant provided informed verbal consent to participate in this study.

## Author contributions

JL: Conceptualization, Data curation, Formal analysis, Funding acquisition, Investigation, Methodology, Project administration, Software, Validation, Visualization, Writing – original draft, Writing – review & editing, Resources. WJ: Formal analysis, Methodology, Project administration, Supervision, Validation, Visualization, Writing – review & editing. HH: Conceptualization, Formal analysis, Funding acquisition, Investigation, Methodology, Project administration, Supervision, Validation, Visualization, Writing – original draft, Writing – review & editing.
